# Localization of Epileptogenic Zone With the Correction of Pathological Networks

**DOI:** 10.3389/fneur.2018.00143

**Published:** 2018-03-14

**Authors:** Chuanzuo Yang, Guoming Luan, Qian Wang, Zhao Liu, Feng Zhai, Qingyun Wang

**Affiliations:** ^1^Department of Dynamics and Control, Beihang University, Beijing, China; ^2^Beijing Key Laboratory of Epilepsy, Sanbo Brain Hospital, Capital Medical University, Beijing, China; ^3^Department of Neurosurgery, Epilepsy Center, Sanbo Brain Hospital, Capital Medical University, Beijing, China; ^4^Beijing Institute for Brain Disorders, Beijing, China

**Keywords:** refractory focal epilepsy, stereo-electroencephalograph, epileptogenic zone localization, coupled neuronal population model, epileptogenic networks

## Abstract

Patients with focal drug-resistant epilepsy are potential candidates for surgery. Stereo-electroencephalograph (SEEG) is often considered as the “gold standard” to identify the epileptogenic zone (EZ) that accounts for the onset and propagation of epileptiform discharges. However, visual analysis of SEEG still prevails in clinical practice. In addition, epilepsy is increasingly understood to be the result of network disorder, but the specific organization of the epileptic network is still unclear. Therefore, it is necessary to quantitatively localize the EZ and investigate the nature of epileptogenic networks. In this study, intracranial recordings from 10 patients were analyzed through adaptive directed transfer function, and the out-degree of effective network was selected as the principal indicator to localize the epileptogenic area. Furthermore, a coupled neuronal population model was used to qualitatively simulate electrical activity in the brain. By removing individual populations, virtual surgery adjusting the network organization could be performed. Results suggested that the accuracy and detection rate of the EZ localization were 82.86 and 85.29%, respectively. In addition, the same stage shared a relatively stable connectivity pattern, while the patterns changed with transition to different processes. Meanwhile, eight cases of simulations indicated that networks in the ictal stage were more likely to generate rhythmic spikes. This indicated the existence of epileptogenic networks, which could enhance local excitability and facilitate synchronization. The removal of the EZ could correct these pathological networks and reduce the amount of spikes by at least 75%. This might be one reason why accurate resection could reduce or even suppress seizures. This study provides novel insights into epilepsy and surgical treatments from the network perspective.

## Introduction

Focal epilepsy is characterized by paroxysmal abnormal discharge within local brain tissue, approximately 30% of which are medically refractory (1, 2). Drug-resistant epilepsy is defined as recurrent seizures despite adequate trials of at least two antiepileptic drugs (3, 4). For these patients, the surgical resection can be an effective way by which to achieve seizure freedom. However, the rather high rate of failure in epilepsy surgery underlines that the accurate localization of the epileptogenic area is still intractable. It is generally believed that the epileptogenic zone (EZ) acts as the driving hub of abnormal activity and sustains the seizures. Therefore, one of the purposes of research is to verify that causal analysis can assist in the localization. Furthermore, epilepsy is increasingly understood as the result of network disorder. This study also intends to confirm the existence of epileptogenic connectivity patterns and investigate the influence of resection on these pathological networks.

Patients with refractory focal epilepsy require long-term electroencephalograph, functional imaging (fMRI, PET, ictal SPECT, MRS, or MEG) and neuropsychological testing during the preoperative period (5). When data obtained from the above metrics cannot reach a concordant conclusion, invasive investigations must be done, particularly stereo-electroencephalograph (SEEG) which represents the “gold standard” for the EZ localization (6). However, the EZ identification still depends on the visual inspection of SEEG in clinical practice and is inevitably restrained by subjectivity and ambiguity (7). In recent years, considerable attention has been given to those methods, which aim at quantifying interactions between neuronal populations, since it is well assumed that epileptic phenomena are associated with changes in the brain network (8, 9). Directed transfer function, a multivariate directional connectivity measure, was designed to reveal the causal relationships (10). Adaptive directed transfer function (ADTF) was then proposed because conventional method could not handle non-stationary signals (11, 12).

In previous studies, ADTF was widely used to analyze the ictal discharge or the interictal spike data, while little attention was given to the interictal segments without spikes. Wilke et al. applied the interictal spike recordings to investigate the origin of abnormal discharge (13). Mierlo et al. focused on elaborate connectivity patterns during the first 20 s of clinical seizures (14). Graph measures (e.g., degree and betweenness centrality) were found to correlate with epileptogenic focus (9, 14, 15). However, there is no comparison between the networks from different stages, and the evolution process is not revealed. In this study, we used long-term recordings involving the interictal, pre-ictal, and ictal stages to localize the EZ and demonstrate changes in the connectivity patterns. By comparing with the clinical conclusions, the accuracy and detection rate are calculated to describe the performance of this technique.

In addition, the connectivity pattern in the ictal process may qualitatively differ from others. After all, it is closely associated with seizures. To verify whether networks in the ictal stage are more likely to generate spikes, computational models are adopted. Previous studies were committed to building a bi-stable system, where fixed point represented resting state and limit cycle described the seizure-like dynamics (16, 17). However, the physiological significance of these model parameters was unclear. Comparatively, Wendling’s neural mass model was developed on the basis of physiological discovery and could interpret various events at the neuronal population level (18, 19). Such computational model simulates the dynamics of single node in the network. When multiple models are coupled together, network behaviors can be demonstrated. All connections derive from the causal analysis of SEEG, which differs from applying structural network or functional connectivity (20, 21). Furthermore, by removing individual populations in the coupled model, virtual surgery could be operated to investigate the influence of resection on the networks. It may provide new insights into the relationships between the EZ and epileptogenic networks.

In this study, we apply the ADTF technique to SEEG recordings from 10 patients with focal drug-resistant epilepsy. Dynamic effective connectivity is constructed and out-degree is selected to identify the epileptogenic focus. The calculated results are compared with the clinical conclusions. Furthermore, we establish coupled neuronal population model to simulate electrical activity in various conditions (no resection, random resection, and the removal of the EZ). The outcomes of the virtual resection are then demonstrated, and the relationships between the EZ and pathological network are discussed.

## Materials and Methods

### Clinical Data

The SEEG dataset was obtained from 10 patients (5 males) at Sanbo Brain Hospital of Capital Medical University in Beijing. All were selected based on the following criteria: refractory focal epilepsy and neurosurgical resection rendering the patient seizure-free during at least 2 years. This study was carried out in accordance with the recommendations of the Ethics Committee of the Sanbo Brain Hospital of Capital Medical University with written informed consent from all subjects. The basic characteristics of these recruited patients are described in Table 1. The age is presented as a range to avoid identifiable patient information.

**Table 1:** Clinical patient characteristics.

Patient	Age (years)	Duration (years)	Side	Electrodes/contacts	Recorded seizures	Pathology
1	16–20	12	R	15/124	4	FCD Ia
2	6–10	5	L	11/116	6	FCD Ib
3	≤5	7/12	R	13/122	9	FCD IIa
4	6–10	1	L	10/120	101	FCD IIb + FCD Ic
5	≤5	3	R	10/108	2	FCD Ib
6	16–20	3	L and R	15/119	4	FCD Ib + FCD IIb
7	30–35	22	L and R	9/126	9	HS
8	6–10	5	L	13/116	1	FCD Ib
9	26–30	12	L	8/108	5	HS
10	10–15	9	R	8/117	17	FCD Ib + GMH

*FCD, focal cortical dysplasia; HS, hippocampus sclerosis; GMH, gray matter heterotopia*.

The locations of electrodes were decided on the findings of non-invasive investigations, and the implanting operation aided by an advanced robot ROSA was completed accurately. Each electrode had multiple contacts, each of which was regarded as a channel. In addition, the average monitoring period was 1 week, and the sampling frequency was set to 512 Hz.

For each patient, we randomly selected two episodes (patient No. 8 had only one) containing epileptic seizures. These episodes were taken from 60 s before the pre-ictal stage to 30–60 s after seizure onset. It is worth mentioning that the pre-ictal period (IP) was divided due to the presence of high-frequency oscillations, also referred to as rapid discharges (22). If patients did not have a definite pre-ictal stage, the time series would begin at 60 s before clinical seizure.

### Adaptive Directed Transfer Function

The Granger causality is a classical technique to evaluate the statistical interdependence of multiple simultaneous time series (23), the strength of which can be quantified using multivariate autoregressive model (MVAR). To track the properties of non-stationary SEEG, the model parameters should be adaptive rather than fixed. Consequently, time-variant autoregressive model (TVAR) can be represented by the following equation:

(1) $$X\left(n\right)=\sum_{i=1}^{p}A_{i}\left(n\right)X\left(n-i\right)+E\left(n\right),$$

where *X*(*n*) = [*x*_1_(*n*), …, *x_k_*(*n*)]*T* is a vector indicating the recordings of *K* channels at time *n, A_i_*(*n*) is the *K* × *K* coefficient matrix for delay *i* relative to time *n, E*(*n*) is the prediction error assumed to be white noise, and *p* is the model order which can be estimated by Akaike information criteria (24).

**Figure 1:**
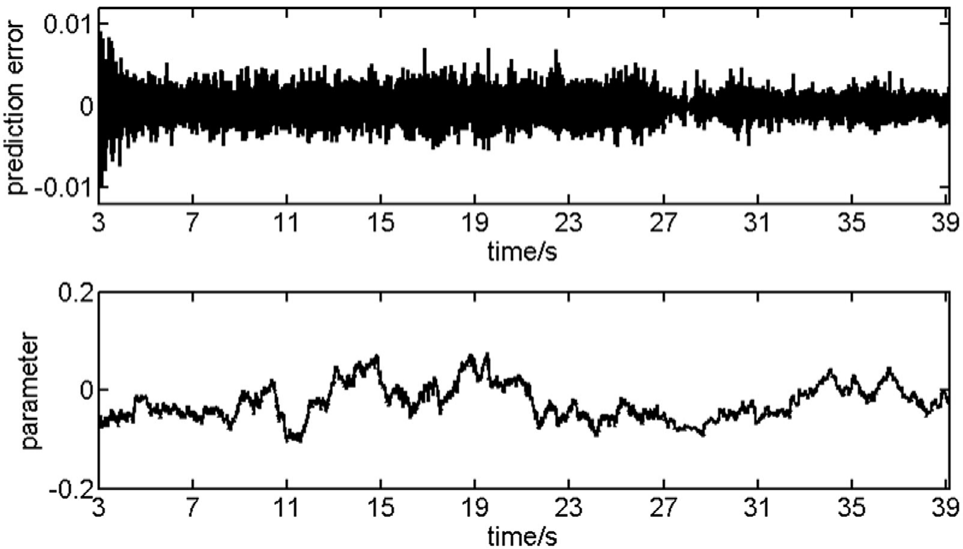
The recordings of 124 channels are used for testing the performance of Kalman filtering algorithm. Result in the first 3 s is abandoned considering the process of adaption.

In fact, the identification of the time-variant coefficient matrix is an ill-posed mathematical issue. Ordinary methods (e.g., least squares, maximum likelihood) are unable to solve it unless time windows are adopted, which may spoil the data. Kalman filtering algorithm based on the state space model is entirely different and can estimate the coefficient matrix at any time. The details of algorithm implementation were described in Arnold et al. (11). An example is presented in Figure 1, wherein the prediction error and the fluctuation of a coefficient are depicted.

At time *n*, the *p* matrices jointly determine the causality relationships between different channels, and ADTF is then developed to integrate these into the frequency domain (25)

(2) $$A\left(f,n\right)=I-\sum_{i=1}^{p}A_{i}\left(n\right)e^{-u.2\pi fi},$$

(3) $${\text{ADTF}}_{ij}\left(f,n\right)={\left|H_{ij}\left(f,n\right)\right|}^{2},$$

where *u* is the imaginary unit, *A* (*f, n*) is the Fourier transformation of the coefficient matrix, and *H_ij_* (*f, n*) is the element of *H* (*f, n*), the inverse matrix of *A* (*f, n*).

Since both the frequency and time can determine the value of *H_ij_* (*f, n*), it may be more convenient to investigate the evolution with time if the information is integrated in a specific frequency band. Meanwhile, the ADTF is generally normalized with respect to the incoming flow, which leads to the spectrum-weighted ADTF (swADTF) (26)

(4) $${\text{swADTF}}_{ij}\left(n\right)=\sum_{f=f_{1}}^{f_{2}}\frac{{\left|H_{ij}\left(f,n\right)\right|}^{2}\sum_{l=1}^{K}{\left|H_{jl}\left(f,n\right)\right|}^{2}}{\sum_{k=1}^{K}\sum_{f\prime =f_{1}}^{f_{2}}\left({\left|H_{ik}\left(f\prime ,n\right)\right|}^{2}\sum_{s=1}^{K}{\left|H_{ks}\left(f\prime ,n\right)\right|}^{2}\right)}.$$

The swADTF is calculated in the frequency band (3, 45 Hz) to exclude the interference of background electrical activity and include the effect of low gamma rhythm simultaneously. Notably, the values of swADTF have a slight fluctuation even for stationary signals. Therefore, it is necessary and reasonable to take the average value every 0.5 s.

### Dynamic Effective Connectivity

**Figure 2:**
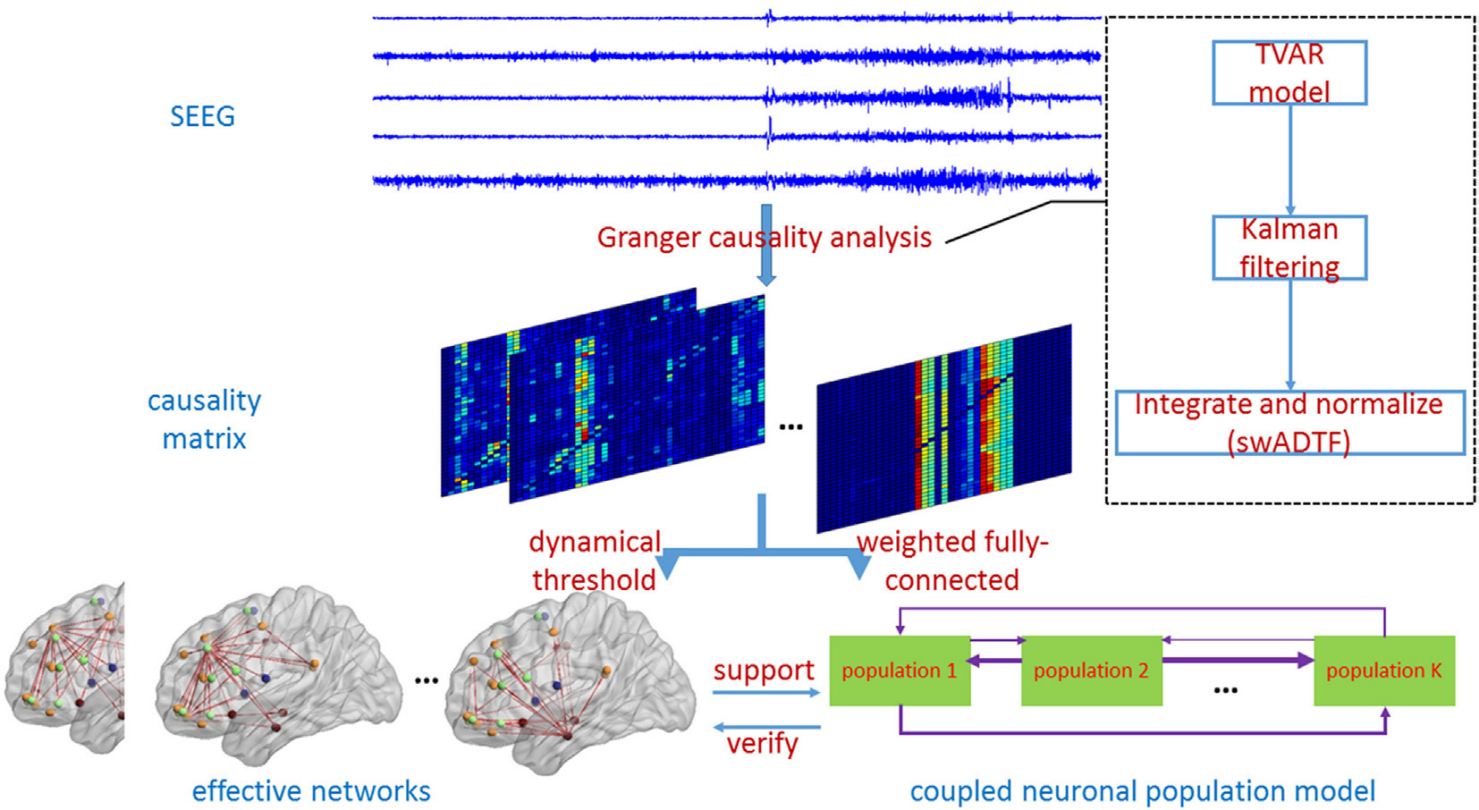
The schematic diagram illustrates the general framework of methods in this study. Granger causality analysis consists of three steps in the dotted box. Later, the left branch shows the procedures of establishing dynamic effective connectivity. Coupled neuronal population model used for simulations is on the right. Furthermore, two branches are interrelated and unified.

Effective connectivity is defined as the influence that one node exerts over another, In contrast to functional connectivity, it can describe the directionality of interactions and the path of information flow. The abnormal activity of the epileptogenic area can stimulate the connected neurons and cause sporadic or periodic spikes. The EZ often maintains robust causality relationships with neuronal populations. Therefore, the top *K* non-diagonal elements of the causality matrix are selected to determine the dynamic threshold th (*n*). The connection matrix *L* is defined as follows:

(5) $$\{\begin{array}{l} {\text{swADTF}}_{ij}\left(n\right)\geq \text{th}\left(n\right)\text{ and }i\neq j\to L_{ij}\left(n\right)=1\\ {\text{swADTF}}_{ij}\left(n\right)\leq \text{th}\left(n\right)\text{ or }i=j\to L_{ij}=0 \end{array},$$

where *L_ij_*(*n*) = 1 indicates that there exists an edge from node *j* pointing to node *i*. The process of constructing dynamic effective connectivity is described in the left branch of Figure 2.

The out-degree is the number of outgoing edges from one node. A large out-degree means that this node dominated the network at that time. Its role is extremely similar to the impact of the EZ on other neural ensembles in the production of synchronous discharges. Therefore, the out-degree is selected as the principal indicator of the epileptic area.

### Coupled Neuronal Population Model

**Figure 3:**
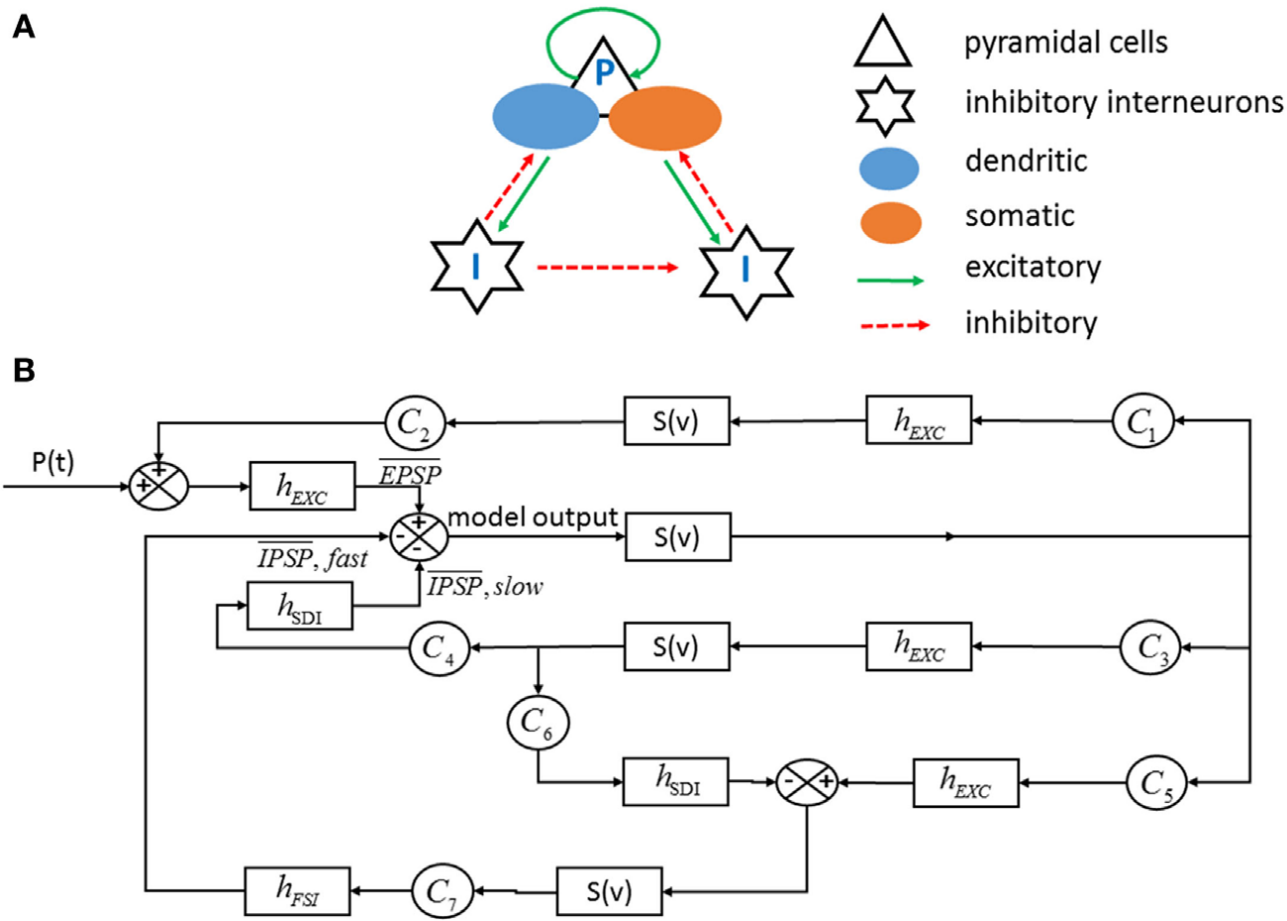
**(A)** The structure of single neuronal population includes three subsets of cells and excitatory and inhibitory connections. **(B)** Corresponding block diagram representation. *S*(*v*) model the saturation effects resulted from average pulse density of action potentials. *h*_EXC_, *h*_SDI_, and *h*_FSI_ are the impulse response, respectively, determining the excitatory, the dendritic inhibitory, and the somatic inhibitory average presynaptic potentials. *p*(*t*) globally measures the influence of neighboring or distant populations, which is assumed to be Gaussian white noise. *C*_1_, *C*_2_, …, *C*_7_ are the average number of synaptic contacts in the feedback excitatory or inhibitory loop [adapted from Wendling F and Chauvel P with permission from Elsevier (31)].

In 2002, Wendling et al. proposed an exquisite single neuronal population model on the basis of previous research results (18, 27, 28). In this model, physiological insights have been considered as follows: (i) the non-uniform alteration of GABAergic inhibition in experimental epilepsy (29); (ii) the possible depression of GABA_fast_ circuit activity by GABA_slow_ inhibitory postsynaptic currents (30). This model includes three subsets of neurons, namely the main cells (e.g., pyramidal cells), the dendritic-projecting inhibitory interneurons (GABA_A,slow_ receptors) and the somatic-projecting inhibitory interneurons (GABA_A,fast_ receptors). Excitatory and inhibitory connections between subsets are depicted in Figure 3A, and their intrinsic mechanism is described in the form of block diagram (Figure 3B).

**Figure 4:**
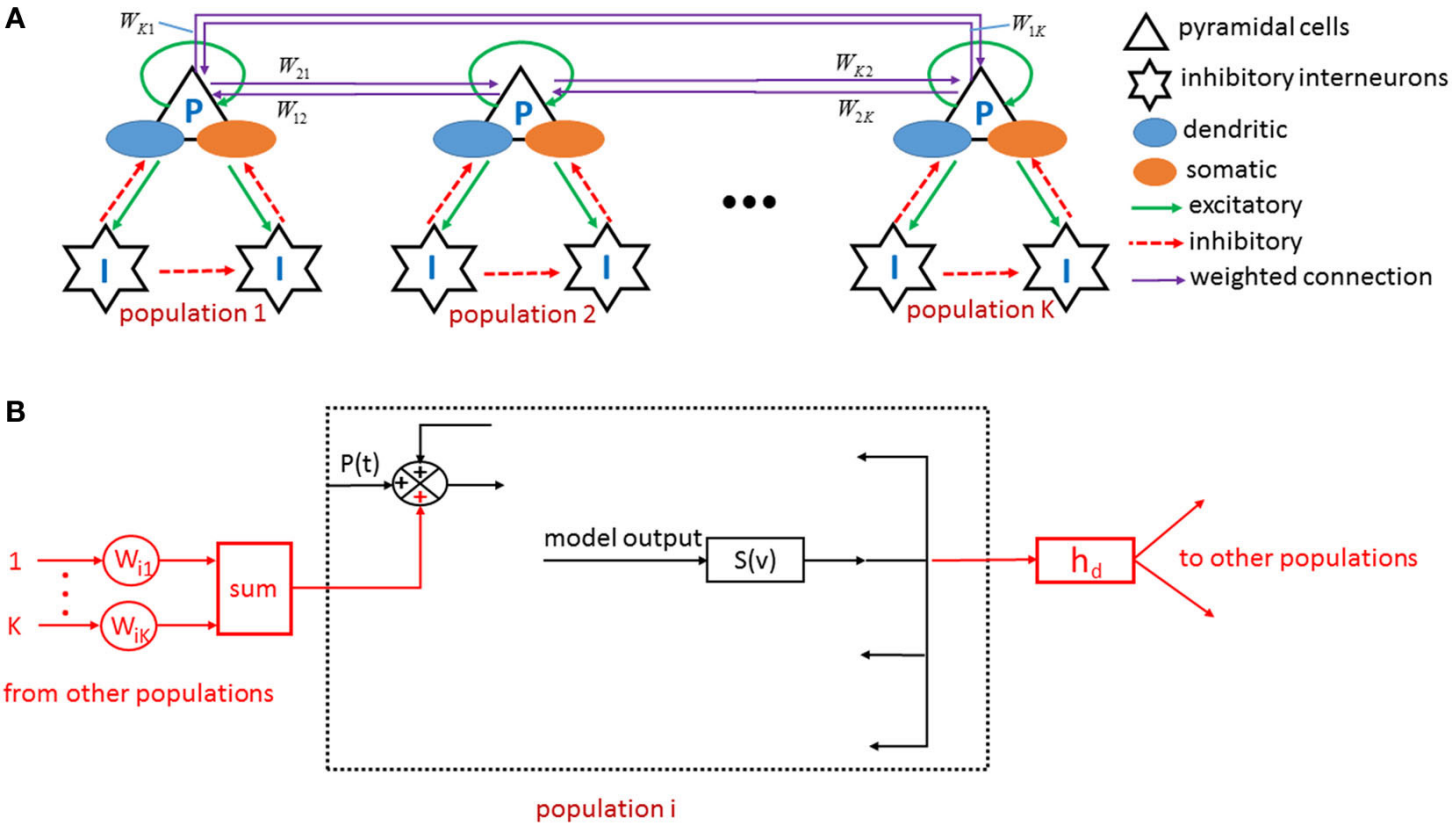
**(A)** Neuronal populations are coupled by directional weighed edge to come into being a fully connected network. **(B)** The generation of input and output signals is depicted by taking an example of population *i*, the internal structure of which is shown in Figure 3B. *h_d_* is an impulse response function similar to *h*_EXC_, *h*_SDI_, and *h*_FSI_.

Neuronal populations are connected and cooperate to achieve specific functions. To account for this organization, a coupled neuronal population model is established (Figure 4), and the connection strength is represented by the following equation:

(6) $$\{\begin{array}{l} \begin{array}{cc} W_{ij}\left(n\right)=\gamma {\text{swADTF}}_{ij}\left(n\right); & i\neq j \end{array}\\ \begin{array}{cc} W_{ij}=0; & i=j \end{array} \end{array}$$

where γ is the amplification factor that neutralizes the impact of normalization. Its value depends on the size of the network, which generally takes 320–360.

According to the block diagram, the following set of 12 × *K* differential equations that describe the state of every population can be established:

(7) $$\begin{array}{l} \left\{ \begin{array}{l} {\dot{y}}_{1}^{i}=y_{6}^{i}\\ {\dot{y}}_{6}^{i}=\text{EXC}\cdot aS\left(y_{2}^{i}-y_{3}^{i}-y_{4}^{i}\right)-2ay_{6}^{i}-a^{2}y_{1}^{i}\\ {\dot{y}}_{2}^{i}=y_{7}^{i}\\ {\dot{y}}_{7}^{i}=\text{EXC}\cdot a\left[p^{i}\left(t\right)+C_{2}S\left(C_{1}y_{1}^{i}\right)+\sum_{j=1,j\neq i}^{k}W_{ij}y_{11}^{i}\right]-2ay_{7}^{i}-a^{2}y_{2}^{i}\\ {\dot{y}}_{3}^{i}=y_{8}^{i}\\ {\dot{y}}_{8}^{i}=\text{SDI}\cdot bC_{4}S\left(C_{3}y_{1}^{i}\right)-2by_{8}^{i}-b^{2}y_{3}^{i}\\ {\dot{y}}_{4}^{i}=y_{9}^{i}\\ {\dot{y}}_{9}^{i}=\text{FSI}\cdot gC_{7}S\left(C_{5}y_{1}^{i}-C_{6}y_{4}^{i}\right)-2gy_{8}^{i}-g^{2}y_{4}^{i}\\ {\dot{y}}_{5}^{i}=y_{10}^{i}\\ {\dot{y}}_{10}^{i}=\text{SDI}\cdot bs\left(C_{3}y_{1}^{i}\right)-2by_{10}^{i}-b^{2}y_{5}^{i}\\ {\dot{y}}_{11}^{i}=y_{12}^{i}\\ {\dot{y}}_{12}^{i}=\text{EXC}\cdot a_{d}S\left(y_{2}^{i}-y_{3}^{i}-y_{4}^{i}\right)-2a_{d}y_{12}^{i}-a_{d}^{2}y_{11}^{i} \end{array}\right.\\ i=1,2\ldots K. \end{array}$$

The parameters’ interpretation and standard values are listed in Table 2 (18, 28). In the process of simulation, it is assumed that all populations have the same parameters values so that only the relationship between seizures and networks is investigated.

**Table 2:** Model parameters, interpretation, and standard values.

Parameter	Interpretation	Standard value
EXC	Excitatory synaptic gain	3.25 mV
SDI	Slow inhibitory synaptic gain	22 mV
FSI	Fast inhibitory synaptic gain	20 mV
1/*a*	Time constant in the feedback excitatory loop	*a* = 100 s*^−^*^1^
1/*b*	Time constant in the slow feedback inhibitory loop	*b* = 50 s*^−^*^1^
1/*g*	Time constant in the fast feedback inhibitory loop	*g* = 500 s*^−^*^1^
1/*a_d_*	Time constant associated to connections	*a_d_* = 30 s*^−^*^1^
*C*_1_, *C*_2_	Average number of synaptic contacts in the slow feedback inhibitory loop	*C*_1_ = *C, C*_2_ = 0.8*C* (with *C* = 135)
*C*_3_, *C*_4_	Average number of synaptic contacts in the slow feedback inhibitory loop	*C*_3_ = 0.25*CC*_4_ = 0.25*C*
*C*_5_, *C*_6_	Average number of synaptic contacts in the fast feedback inhibitory loop	*C*_5_ = 0.3*CC*_6_ = 0.1*C*
*C*_7_	Average number of synaptic contacts between slow and fast inhibitory interneurons	*C*_7_ = 0.8*C*

It is also possible to perform virtual surgery and observe outcomes by removing the specified population from the model. For instance, if population *u* is selected for removal, the coupling matrix *W*(*n*) can be changed into *W*′(*n*)

(8) $$\{\begin{array}{l} \begin{array}{ccc} W_{ij}^{\prime }\left(n\right)=W_{ij}\left(n\right);i\neq u & \text{and} & j\neq u \end{array}\\ \begin{array}{ccc} W_{ij}^{\prime }\left(n\right)=0;i=u & \text{or} & j=u \end{array} \end{array}$$

Thus, it is easy to investigate the outcomes of different operation schemes, including the removal of the EZ based on theoretical calculation.

## Results

### Localizing the EZ

The comparison between the out-degree calculation results and clinical conclusions is shown in Table 3. The electrode contacts with the maximum out-degree in various stages are regarded as the theoretical epileptogenic focus. SEEG reports accomplished by several experienced electrophysiologists indicate which channels are involved in the onset of epileptiform discharges. All of the epileptogenic channels in the SEEG reports are located in the resection region. Fine surgical outcomes can ensure that the clinical conclusions are credible enough to evaluate the theoretical results. In addition, the pre-ictal stage is combined with the ictal process since it has long been recognized as the sign of seizure onset (22).

**Table 3:** Calculated results compared with clinical conclusions.

No.	Calculated result		Stereo-electroencephalograph report		Surgery region			
		
	IIP	IP	IIP	IP	A.B.	P.B.	S.B.	I.B.
1	D02	D11, D12	D11	D12	MTG	RF. of CG. on L.Sur.	SMG. to IPS	BT
2	E04	E04, J14, M08	E04	E04, J13, M05	6 cm before PreCS.	PreCS.	L.Sur.	IFS. to CC.
3	P08, E07	E07, M04	P08, E07	E07, G06	CS.	PoCS.	L.Sur.	TSF.
4	L11, H13, I11	L09, I13, H13	L11, H13	L09, H13	2 gyri before PreCS.	PreCS.	L.Sur.	SF.
5	F08, D03	D03	F08, D03	D03	FP.	CSut.	L.Sur.	SF.
6	L04	L04	L04, H04	L04	FP.	CSut.	SFS.	RG.
7	E10	C02, D01	E10	C02, D01	Residual tissue	5.5 cm to TP	Hippo.	Hippo.
8	G10	G10	G10	G10, H10	3 cm to TP	6 cm to TP	SF.	STS.
9	A01, B01	A01, B01, E03	A01, B01	A01, B01	SAH. (amygdala + hippocampus head and 3 cm tail)			
10	K09, G11	K09	K09, G11	K09, G11	FP.	PreCS.	L.Sur.	SF.

*IIP, interictal period; IP, ictal period; A.B., anterior boundary; P.B., posterior boundary; S.B., superior boundary; I.B., inferior boundary; BT., basal surface of temporal lobe; CC., corpus callosum; CG., cuneus gyrus; CS., central sulcus; CSut., coronal suture; FP., frontal pole; Hippo., hippocampus; IFS., inferior frontal sulcus; IPS., inferior parietal sulcus; L.Sur., lateral surface; MTG, medial temporal gyrus; PreCS., precentral sulcus; PoCS., postcentral sulcus; RF., reflection; RG., rectal gyrus; SF., sylvian fissure; SFS., superior frontal sulcus; SMG., superior marginal gyrus; STS., superior temporal sulcus; TP, temporal pole; TSF., tail of sylvian fissure*.

The accuracy is the proportion of true positives to all calculated results. The detection rate measures corresponding percentage in clinical conclusions. They describe the influence of false positives and false negatives, respectively. According to Table 3, SEEG reports list 34 epileptogenic channels, while the calculated results show 35, 29 of which are actual positives, meaning that the accuracy is 82.86%, and the detection rate is 85.29%. In addition, the results from eight patients demonstrate that the driving hubs (the channels with the maximum out-degree) of the interictal activity overlap with those in the ictal stage. This implies that the cause of seizures may lurk in the interictal activity.

**Figure 5:**
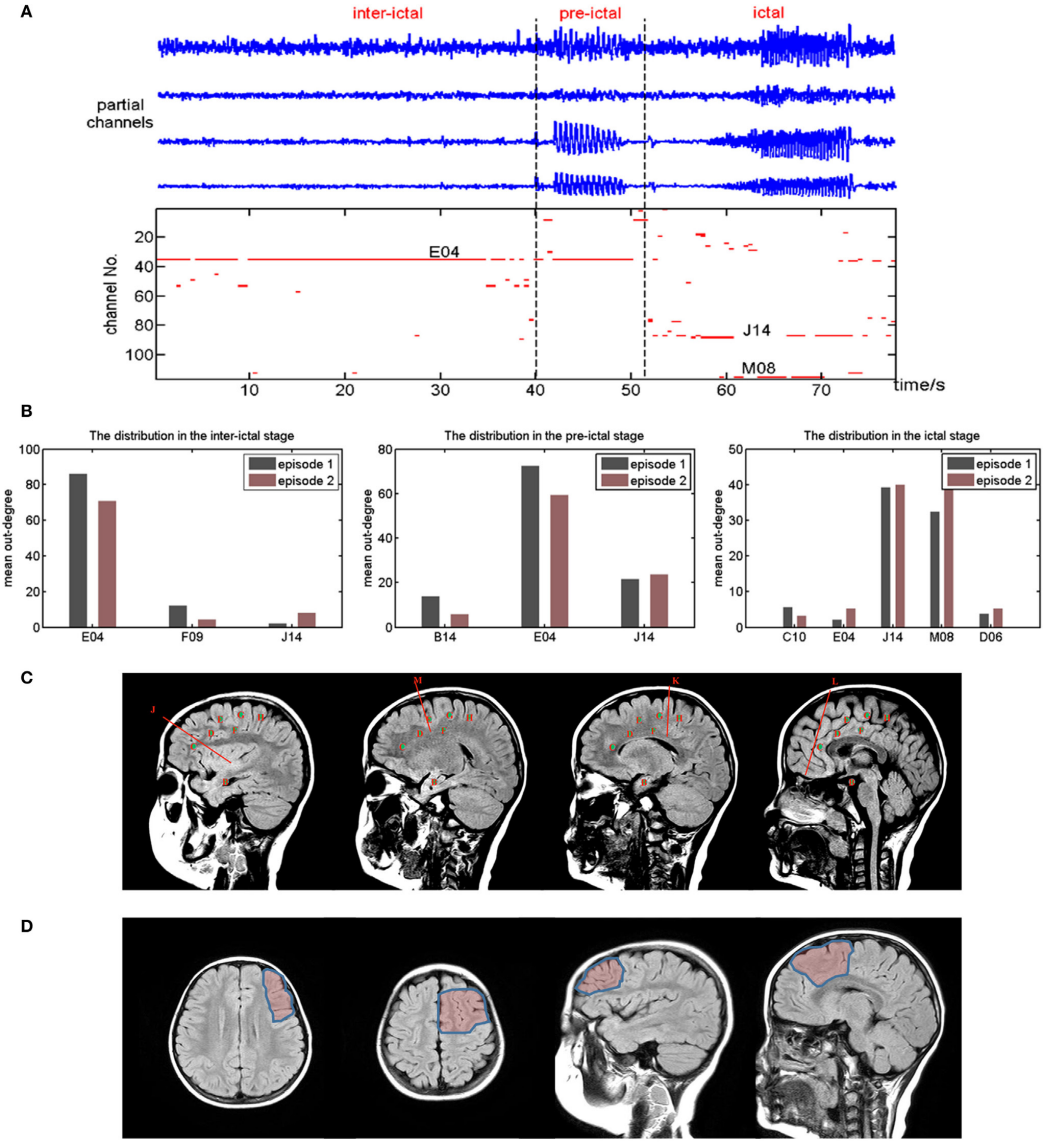
**(A)** The upper is a segment of stereo-electroencephalograph data from partial channels. The bottom is the out-degree of each channel with time (only those above 30 are shown). **(B)** The distribution of mean out-degree in different stages. **(C)** Diagram of electrodes location for patient No. 2. **(D)** Diagram of surgery resection region for patient No. 2.

Particularly, the out-degree of each channel with time is depicted for patient No. 2 in Figure 5A. The stages are divided according to the features of the SEEG data and clinical symptoms. Although the salient characteristics of the brain networks are relatively stable within a certain stage, they differ from one stage to another. To clearly reveal the transient process, a compromise is struck. Only the dots whose out-degree is above 30 are marked. Meanwhile, the networks in the same stage are summed and then averaged. The distribution of the mean out-degree is shown to interpret the difference of connectivity patterns (Figure 5B). In addition, the results are in good agreement during the two episodes.

It is shown that E04, the fourth contact of electrode E, governs the whole networks in both the interictal stage and the pre-IP. Similarly, J14 and M08 dominate activities in the ictal stage. The SEEG report verifies that intermittent spikes can be observed in E04 during the interictal period (IIP). Electrophysiologists deem that the abnormal discharges spread from E04 to electrode J and M. The calculated results are included within the resection area (Figures 5C,D). Fine surgical outcome also proves that this method is effective and accurate for localizing the EZ.

### The Correction of Pathological Networks

**Figure 6:**
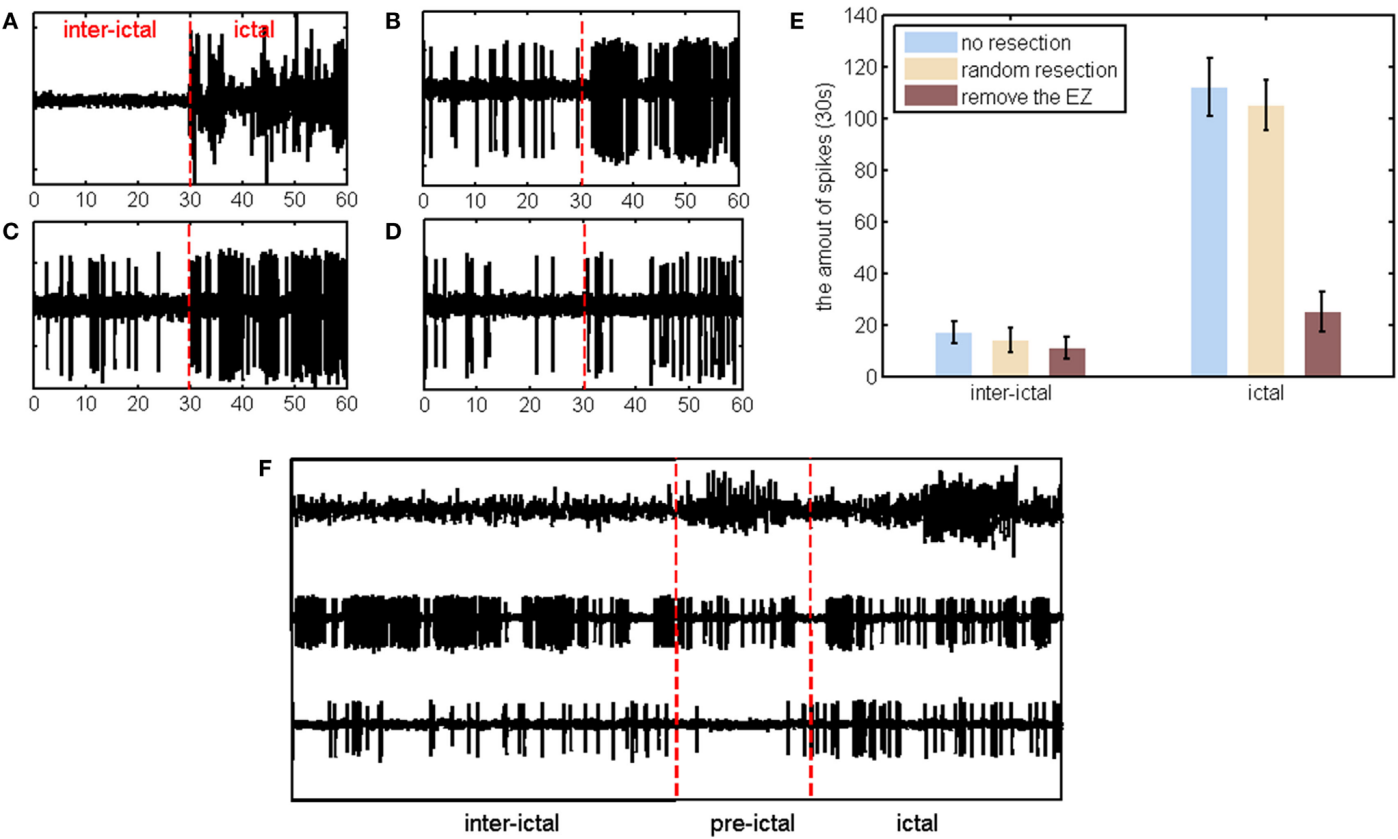
**(A)** Real data of a channel for patient No. 1. The left is in the interictal stages, and the right is in the ictal process without evident pre-ictal stage. **(B)** Corresponding simulation result based on coupled neuronal population model. **(C)** Simulation result after removing one channel outside the epileptogenic zone (EZ). **(D)** Simulation result after removing one channel inside the EZ. **(E)** The amount of spikes in three conditions (no resection, random resection, and removing the epileptogenic channel) during the simulation process. **(F)** Stereo-electroencephalograph recording, simulation without resection and simulation after removing one channel inside the EZ for patient No. 2.

Spikes are one of the most common biomarkers for indicating abnormal brain activity. In the simulation process, sporadic spikes are seen in the interictal stage and rhythmic spikes are observed during the IP (Figure 6B), which are consistent with clinical findings (Figure 6A). Virtual surgical resections are then carried out, and the outcomes of different operations are observed (Figures 6C,D). These simulations in three conditions (no resection, random resection, and removing the epileptogenic channel) are repeated 30 times, respectively. Compared with random resection, the removal of the EZ is more effective to reduce the amount of spikes (at least 75%) (Figure 6E). It is worth mentioning that the clinical resection range is wider than single channel, so sporadic spikes can be further eliminated.

However, two cases of simulations are not in accordance with the real situation. In Figure 6F, periodic spikes occupy almost the entire IIP, while there are only partial spikes during the ictal process. Nevertheless, the channels with the maximum out-degree in the interictal stage were also the targets for surgical resection. These spikes can be eliminated after removing the calculated source. Such interictal networks are also pathological and may well be the cause of seizures.

## Discussion

This study developed a framework to analyze long-term SEEG data and localize the epileptogenic area. The electrode contacts with the highest total out-degree corresponded well with epileptogenic region defined by electrophysiologists. The same stage shared a relatively stable connectivity pattern, while the patterns varied from stages. Coupled neuronal population model was established to investigate the presence of epileptogenic patterns. Simulations indicated that most networks during the IP were epileptogenic, and the nature of epileptogenic network was to enhance local excitability and facilitate synchronization. The fact that removing the EZ could significantly reduce the amount of spikes suggested that the EZ should be responsible for the formation of these pathological networks.

### The Value of the Interictal Process

Epilepsy is regarded as a network-level phenomenon (32). This type of network disorder may have already existed before clinical seizures. Intermittent spikes during this period can be regard as implications. Previous studies have demonstrated that interictal epileptic events were helpful for confirming the range of abnormal areas (33, 34). Wilke et al. concluded that the sources of interictal spike activity might be the generators underlying the clinical seizures (13). In Table 3, eight patients demonstrated that the sources of the interictal activity overlapped with the locations that the ictal activity originated from. In addition, some networks in the interictal stages exhibited epileptogenic behaviors and rhythmic spikes were observed in the simulations. This organization that is prone to producing spikes may push brain function to the edge of disorder. In other words, eliminating the interictal spikes or correcting these pathological networks should be part of epilepsy treatment.

### Epileptogenic Networks

It is increasingly understood that seizures arise from epileptogenic networks, through which epileptiform discharges are produced and propagated (35). However, it is still unclear that which type of network can be considered as epileptogenic connectivity. Epileptogenic networks are not equal to the networks in the ictal stage. Some networks in the interictal stage were also epileptogenic. Varotto et al. reached a similar conclusion on the basis of SEEG recordings from patients with type II focal cortical dysplasia (36). Brain functional connectivity identified by the fMRI also showed an abnormal pattern in the absence of epileptiform discharges (37).

We have known that the channel with the maximum out-degree varies from stages. However, the occurrence of seizures is not only due to the transfer of important nodes. Qualitative change of connectivity patterns must follow. Coupled neuronal population model was used to simulate the time course in different patterns. An increased number of spikes indicate that this connectivity pattern is more likely to generate ictal activity. The nature of epileptogenic network should be a specific organization which likely enhances local excitability and facilitates synchronization. Because the epileptogenic region affects other neural ensembles and enables them to synchronize, it should be responsible for the formation of these pathological networks. Virtual surgery also suggested that the removal of the EZ could significantly reduce the amount of spikes. Sinha et al. also concluded that the resection of these regions could reduce the overall likelihood of seizures after conducting simulations with a bi-stable model (21). Hence, it is helpful to explain why seizures are reduced or suppressed after accurate resection.

### Model Simulation and Virtual Surgery

The distribution of the out-degree could aid identification of the EZ and deepen our understanding of the difference between connectivity patterns. However, it failed to describe the qualitative changes of network organization. Coupled neuronal population model was used to demonstrate the network behavior and judge whether the network was epileptogenic or not. It was also convenient to observe the outcomes of different operation schemes. There are two reasons to carry out virtual surgery. First, contradiction may appear when the EZ overlaps with the brain function area. Hypothetical surgery provides new options for controlling seizures with regard to preserving major function. Second, virtual surgery is beneficial in terms of balancing the resection range and therapeutic effect (38, 39). It is well known that a smaller resection region means lower physiological cost. By removing one or more channels, it is possible to assess the corresponding surgical effect, and the optimal scope can be determined. In a word, the treatment for refractory focal epilepsy should be a comprehensive and systematic project instead of just achieving seizure freedom.

### The Relationships Between the Two Branches

This study consists of the EZ localization and coupled neuronal population model. The former aims at identifying the electrode contacts triggering the abnormal discharge. However, seizures are often associated with abnormal changes in brain synchronization mechanisms. This model is used to find epileptogenic connectivity patterns and evaluate the role of the EZ on pathological network. The results suggested that the presence of the EZ made the network more easily synchronized. Accordingly, if the EZ is removed, pathological network can be corrected and seizures are reduced or suppressed. It is helpful to understand the relationship between the EZ and epileptogenic networks.

## Conclusion

It was shown that channels with the maximum out-degree corresponded to the epileptogenic area after applying the ADTF technique to SEEG recordings from 10 patients. All of these recordings from the interictal, pre-ictal and ictal stages were valuable for the EZ localization. Interestingly, we found that the same stage shared a relatively stable connectivity pattern, while the pattern evolved at different stages. In addition, some of these patterns were verified to be epileptogenic. Model simulations confirmed this and suggested that these pathological networks could be corrected if the EZ was removed. It indicated that the nature of the epileptogenic network should be a specific organization enhancing local excitability and facilitating synchronization.

## Ethics Statement

This study was carried out in accordance with the recommendations of the Ethics Committee of the Sanbo Brain Hospital of Capital Medical University with written informed consent from all subjects. All subjects gave written informed consent in accordance with the Declaration of Helsinki. The protocol was approved by the Ethics Committee of the Sanbo Hospital of Capital Medical University.

## Author Contributions

CY, GL, QW, ZL, FZ, and QYW collected and analyzed these clinical recordings to localize the epileptogenic zone and wrote the paper. CY and QYW built mathematical model and carried out a series of simulations.

## Conflict of Interest Statement

The authors declare that the research was conducted in the absence of any commercial or financial relationships that could be construed as a potential conflict of interest.
